# Melatonin Pre-harvest Treatments Leads to Maintenance of Sweet Cherry Quality During Storage by Increasing Antioxidant Systems

**DOI:** 10.3389/fpls.2022.863467

**Published:** 2022-04-11

**Authors:** Alberto Carrión-Antolí, Domingo Martínez-Romero, Fabián Guillén, Pedro J. Zapata, María Serrano, Daniel Valero

**Affiliations:** ^1^Department of Agro-Food Technology, University Miguel Hernández, Orihuela, Spain; ^2^Department of Applied Biology, University Miguel Hernández, Orihuela, Spain

**Keywords:** *Prunus avium*, phenolics, anthocyanins, firmness, colour, soluble sugars, acidity, antioxidant enzymes

## Abstract

Melatonin has been reported to have an important role in fruit ripening, although the effect of pre-harvest melatonin treatment on sweet cherry quality properties during storage is still unknown. In the present experiments, the effects of melatonin (0.1, 0.3, and 0.5 Mm) by foliar spray treatments of ‘Prime Giant’ and ‘Sweet Heart’ sweet cherry trees on fruit quality traits and antioxidants systems during storage was evaluated. Results showed that these treatments reduced weight losses during storage, as well as losses in firmness and titratable acidity. In addition, changes in fruit colour and total soluble solid content were also delayed in fruit from melatonin treated trees with respect to controls. Moreover, in general, total phenolic and anthocyanin concentrations were higher in fruit from treated trees than in those from control ones, either at harvest or during the whole storage period. Finally, the activity of the antioxidant enzymes catalase, ascorbate peroxidase and peroxidase was also enhanced as a consequence of melatonin treatment. Overall results show that pre-harvest melatonin treatment delayed the post-harvest ripening process of sweet cherry fruit, leading to maintenance of their quality properties in optimum levels for consumption 2 weeks more with respect to fruit from control trees. Antioxidant systems, both enzymatic and non-enzymatic ones, were also enhanced by melatonin treatments, which would account for the delay on fruit post-harvest ripening process and fruit quality maintenance during storage.

## Introduction

Sweet cherry fruit (*Prunus avium* L.) have excellent organoleptic and nutritional properties, such as appearance, colour, texture, flavour, juiciness, and sugar and organic acid content (Usenik et al., 2008; Díaz-Mula et al., 2009; Martínez-Esplá et al., 2014). In addition, sweet cherries are rich in bioactive compounds with antioxidant properties, mainly phenolics, and ascorbic acid, which are responsible for their health beneficial properties, namely, anti-inflammatory, antidiabetic, antimicrobial, and anticancer effects as well as cardiovascular and neuroprotection activities (McCune et al., 2011; Blando and Oomah, 2019; Faienza et al., 2020). However, they are very perishable fruit suffering from quickly quality losses after harvest even under storage in cold conditions. Thus, different post-harvest treatments combined with cold storage have been reported to be useful to maintain sweet cherry fruit quality for longer time, such as alginate coating (Díaz-Mula et al., 2012), *Aloe vera* gel containing rosehip oil (Paladines et al., 2014), nano-silica coating (Meng et al., 2022), 1-methylciclopropene and chlorine dioxide treatments, alone or in combination (Serradilla et al., 2019; Zhao et al., 2021) or salicylic (SA), acetylsalicylic (ASA), and oxalic (OA) acids treatments (Valero et al., 2011), as well as storage under modified atmosphere conditions (Cozzolino et al., 2019), among others (Correia et al., 2017).

In addition, different pre-harvest treatments, such as gibberellic acid (Einhorn et al., 2013), oxalic acid (Martínez-Esplá et al., 2014), salicylic acid (SA), acetyl salicylic acid (ASA), and methyl salicylate (MeSa) (Giménez et al., 2014, 2017; Valverde et al., 2015) have been performed aimed to increase sweet cherry fruit quality attributes at harvest. These treatments led to enhanced fruit size, firmness and total anthocyanin and phenolic contents at harvest and these quality parameters were maintained during storage, leading to fruit with increased shelf life. In addition, the activity of antioxidant enzymes, such as peroxidase (POD), catalase (CAT), ascorbate peroxidase (APX), and superoxide dismutase (SOD), was also enhanced by these salicylate treatments (Valverde et al., 2015; Giménez et al., 2017). These antioxidant enzymes are involved on scavenging reactive oxygen species (ROS) species, such as hydrogen peroxide (H_2_O_2_), superoxide radical (O2^•–^), hydroxyl radical (OH^•^), or ^1^O^2^, which are inevitably generated in normal metabolism of plant cells but accumulated during fruit ripening and senescence, contributing to peroxidation of membrane lipids, damage to DNA and proteins, and acceleration of senescence processes. Thus, treatments aimed to increase the ability of fruit tissues to decrease ROS levels, by enhancing antioxidant enzyme activities and/or antioxidant compounds, such as phenolics or anthocyanins, have been reported to delay ripening and senescence process and, in turn, to maintain fruit quality in a wide range of fruit species including sweet cherry. In this sense, post-harvest treatments of sweet cherry with hexanal or 1-methylcyclopropene led to higher levels of SOD and APX activities during storage as compared with control cherries (Sharma et al., 2010). Similarly, higher antioxidant enzyme activities during storage were found in sweet cherry fruit coated with chitosan (Dang et al., 2010) or after vacuum cooling treatment (He et al., 2013), as well as in sweet cherry fruit from SA, ASA, or MeSa treated trees (Giménez et al., 2015; Valverde et al., 2015).

Recently, melatonin, which was identified in plants in 1995 (Dubbels et al., 1995), is gaining a broad interest as a universal plant signalling molecule having pivotal roles on regulating a wide range of plant physiological processes, with great potential for its application in the horticultural industry (Aghdam et al., 2020; Arnao and Hernández-Ruiz, 2020a; Tiwari et al., 2020). In particular, post-harvest melatonin treatments have been shown to delay fruit ripening in a wide range of fruit species (Xu et al., 2019; Arnao and Hernández-Ruiz, 2020b). For instance, dipping treatment with 0.5 mM melatonin delayed ripening in mangoes, due to inhibition of ABA and ethylene biosynthesis (Liu et al., 2020), as well as in banana fruit (Hu et al., 2017), which were dose-dependent in the range of 0.05–0.5 mM. Accordingly, a delay of ripening has been reported in peaches and nectarines after post-harvest melatonin treatment (Gao et al., 2016; Bal, 2021). However, the effects of melatonin applied as pre-harvest treatment on on-tree fruit ripening and quality traits have been evaluated in a very few papers and different effects have been found depending on concentration, application time and fruit species. Thus, tomato plant treatment with melatonin, applied in the irrigation system led to increases in lycopene and sugar contents, showing acceleration of the fruit ripening process (Liu et al., 2016). However, melatonin treatment by foliar spray of apricot trees did not affect the on-tree ripening process although positive effects were observed on crop yield and fruit quality parameters at harvest, which were maintained during storage, either at chilling or non-chilling temperatures, as compared with apricots from control trees (Abd El-Naby et al., 2019; Medina-Santamarina et al., 2021). Similar effects of melatonin pre-harvest treatments have been reported recently for ‘Mollar de Elche’ pomegranate (Lorente-Mento et al., 2021).

In sweet cherry fruit, post-harvest dipping melatonin treatments have been recently reported to maintain fruit quality during storage throughout a delay of the senescence process in ‘Sunburst’ (Wang et al., 2019), ‘Siah Mashhad’ (Sharafi et al., 2021), ‘Santina,’ and ‘Royal Rainier’ (Miranda et al., 2020) cultivars. On the other hand, melatonin treatment, applied directly to fruit surface during on-tree fruit development delayed fruit ripening in ‘Prime Giant’ cultivar (Tijero et al., 2019). On the contrary, foliar spray treatment with 0.5 mM melatonin 2 and 1 weeks prior to harvest accelerated fruit ripening of sweet cherry ‘Ferrovia’ (Michailidis et al., 2021). In our previous paper, pre-harvest foliar spray with 0.1, 0.3, and 0.5 mM melatonin led to fruit with enhanced quality traits at harvest, such as fruit weight, colour, firmness, total soluble solid content and titratable acidity (Carrión-Antolí et al., 2022). However, as far as we know, there is not available literature regarding the effect of pre-harvest melatonin treatment on the maintenance of sweet cherry fruit quality properties during storage. Thus, the aim of the present experiment was to evaluate the effects of melatonin foliar spray of sweet cherry trees of ‘Prime Giant’ and ‘Sweet Heart’ cultivars on fruit quality parameters, with especial interest on bioactive compounds and the activity of antioxidant enzymes.

## Materials and Methods

### Plant Material and Experimental Design

The experiments were carried out in a commercial field located at Jumilla (Murcia, Spain, UTMX: 463.700 UTMY: 4.268.900) with ‘Prime Giant’ and ‘Sweet Heart’ sweet cherry (*P. avium* L.) cultivars, in 2019 and 2020 years, respectively. ‘Prime Giant’ was planted in January 2012 and ‘Sweet Heart’ in January 2015 and both were grafted onto SL-64 rootstock. Climatic conditions in the crop field were similar for 2019 and 2020 years: mean annual temperatures 15.24 and 15.30°C for 2019 and 2020, respectively, and accumulated rainfall of 357 and 352 mm for 2019 and 2020, respectively. Agronomic practices were similar for both cultivars with fertilisation of 60:30:100 kg ha^–1^ N:P:K and base type open centre pruning. For each cultivar, three blocks of three trees were selected at random for 0 (control), 0.1, 0.3, and 0.5 mM melatonin treatments. Treatments were applied with a manual sprayer machine (3 L per tree) by using freshly prepared melatonin solutions (containing 1 mL L^–1^ Tween as surfactant) at three key points of fruit development (pit hardening, starting of colour changes and 3 days before harvest), according to previous reports (Giménez et al., 2017; Carrión-Antolí et al., 2022). Sweet cherries were harvested according to commercial practices, when reached their commercial ripening stage, based on the soluble solid content and skin colour of each cultivar. About 3 kg of fruit from each treatment and replicate were taken and transported to laboratory in 3 h. Then, lots of 20 fruits, homogenous in colour and size and without visual defects, were performed at random and stored at 2°C and 90% RH. After 0, 7, 14, 21, and 28 days of storage one lot of each replicate and treatment was taken to perform the following analytical determinations.

### Quality Parameter

Fresh weight of each fruit lot was measured at harvest and at each sampling date during storage by using a digital balance KERN 440-35N (Balingen, Germany) and weight losses were expressed as percentage with respect to weight at day 0. Colour was measured with a Minolta colorimeter (CRC200, Minolta Camera Co., Osaka, Japan), at three equidistant points along the equatorial perimeter of each fruit and was expressed as a*/b* ratio by using the CIELab coordinates. Results are the mean ± SE. Fruit firmness was measured independently in each fruit by using a TX-XT2i Texture Analyzer (Stable Mycrosys-tems, Godalming, United Kingdom) equipped with a flat probe. A force to achieve a 5% fruit diameter deformation was applied and fruit firmness was expressed as the relation between the applied force and the travelled distance (N mm^–1^). Results are the mean ± SE. Then, flesh of the 20 fruit of each replicate was cut in small pieces to obtain a homogeneous sample. A ≈50 g sample was used for total soluble solids (TSS) and titratable acidity (TA) measures, in duplicate, after being squeezed through two layers of cotton cloth. TSS in fruit juice were measured by using a digital refractometer (Atago PR-101, Atago Co. Ltd., Tokyo, Japan) and TA by titration of 1 mL of juice, diluted in 25 mL of distilled H_2_O, with 0.1 N NaOH up to pH 8.1 by using an automatic titration system (785 DMP Titrino, Metrohm, Herisau, Switzerland). TSS and TA results are expressed as g 100 g^–1^ and are the mean ± SE. Other 50 g fruit sample was ground under liquid N_2_, and stored at −20°C until total phenolic and anthocyanin concentrations and antioxidant enzyme activities were measured.

### Total Phenolic and Anthocyanin Quantification

Phenolics were extracted by homogenising 5 g of frozen tissue with 10 mL of water:methanol (2:8) containing 2 mM NaF (to inactivate polyphenol oxidase activity and prevent phenolic degradation) in a Ultraturrax homogeniser (T18 basic, IKA, Berlin, Germany). Then, the extracts were centrifuged at 10,000 × *g* for 10 min at 4°C and total phenolics were quantified in duplicate in the supernatant by using the Folin-Ciocalteu reagent as previously described (Díaz-Mula et al., 2009). Results were expressed as mg gallic acid equivalent 100 g^–1^ and are the mean ± SE. Anthocyanins were extracted by homogenising 2 g of fruit sample with 10 mL of methanol/HCl/water (80:1:19, v/v/v) as addressed above. After centrifugation, anthocyanins were quantified in duplicate in the supernatant by reading absorbance at 530 in a spectrophotometer (UNICAM Helios-α, Artisan Technology Group, Champaign, IL, United States). Total anthocyanins were calculated by using cyanidin-3-glucoside molar absorption coefficient of 23,900 L cm^–1^ mol^–1^ and molecular weight of 449.2 g mol^–1^. Results were expressed as mg cyanidin 3-glucoside equivalent 100 g^–1^ and were the mean ± SE.

### Measure of Antioxidant Enzyme Activities

To obtain crude extract of POD, CAT and APX, 5 g of sweet cherry samples were homogenised with 10 mL of phosphate buffer 50 mM, pH 7.0, containing 1 mM ethylen-diamine-tetraacetic acid (EDTA) and 1% (w/v) polyvinylpyrrolidone. Then, the homogenate was centrifuged at 15,000 × *g* for 30 min at 4°C and antioxidant enzyme activities were measured in the supernatant as previously described (Giménez et al., 2017). Briefly, for POD determination, the reaction mixture contained 50 mM phosphate buffer pH 7.0, 14 mM guaiacol, 12 mM H_2_O_2_ and 100 μL of enzymatic extract in a total volume of 3 mL. The increase of absorbance at 470 nm from time 0 to 1 min, due to guaiacol oxidation, was measured and POD activity was expressed as U min^–1^ g^–1^, one enzymatic unit (U) being defined as 0.01 absorbance increase per min. The reaction mixture for CAT activity contained 100 μL of the above extract and 2.9 mL 50 mM phosphate buffer pH 7.0, containing 15 mM H_2_O_2_ and the decrease of absorbance at 240 nm for 1 min due to H_2_O_2_ degradation was measured and CAT activity expressed as U min^–1^ g^–1^, one enzymatic unit (U) being defined as 0.01 absorbance decrease per minute. Finally, the assay mixture for APX quantification contained 50 mM potassium phosphate pH 7.0, 0.5 mM ascorbic acid, 1 mM H_2_O_2_ and 100 μL of crude extract in a final volume of 3 mL. The decrease of absorbance at 290 nm during 1 min was measured and one enzymatic unit of APX (U) was defined as the amount of enzyme that oxidises 1 mmol of ascorbate per minute, and APX was expressed as U min^–1^ g^–1^.

### Statistical Analysis

The field experiments were performed by using three replicates of three trees per treatment for each cultivar in a completely randomised design. Fruit samples from each replicate were taken and used for storage experiment. Experimental data from each cultivar were independently subjected to ANOVA analysis. For each cultivar, sources of variation were treatment and storage time. All analyses were performed with SPSS software package v. 22.0 for Windows (SPSS, 2011). Least significant differences (LSD) at *p* < 0.05 were calculated and values shown in each figure.

## Results

### Fruit Quality Parameters

**FIGURE 1:**
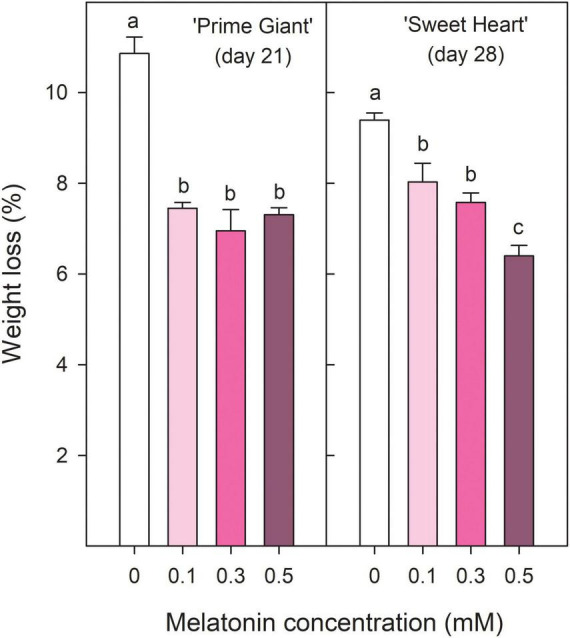
Weight losses for ‘Prime Giant’ and ‘Sweet Heart’ cultivars after 21 and 28 days of storage at 2°C, respectively, as affected by melatonin pre-harvest treatments. Data are the mean ± SE of three replicates of 20 fruits. Different letters show significant differences (*p* < 0.05) among treatments for each cultivar.

**FIGURE 2:**
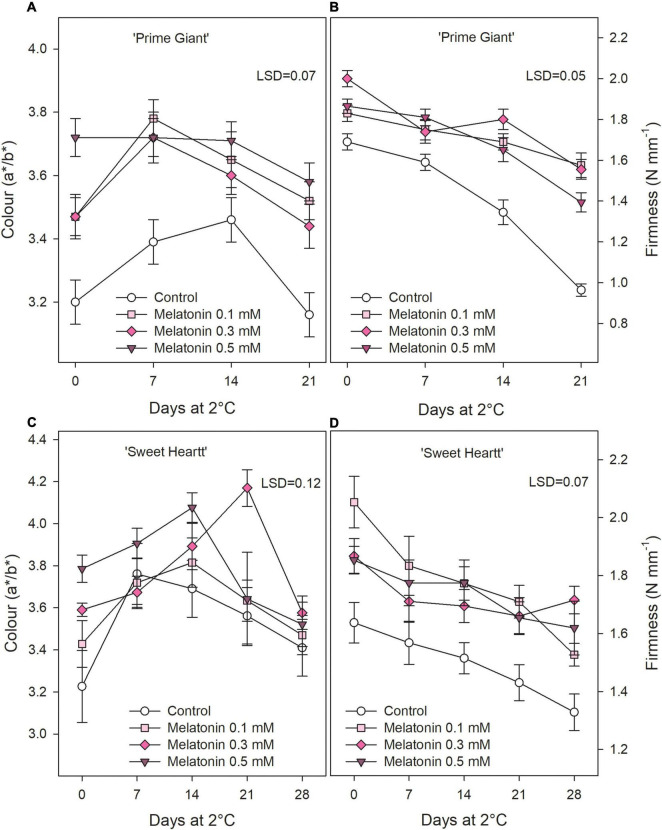
Fruit colour **(A)** and firmness **(B)** for ‘Prime Giant’ and ‘Sweet Heart’ **(C,D)** cultivars during storage at 2°C as affected by melatonin pre-harvest treatments. Data are the mean ± SE of three replicates of 20 fruits. LSD values at (*p* < 0.05) are shown in each figure.

‘Prime Giant’ and ‘Sweet Heart’ cultivars were stored for 21 and 28 days, respectively, until control fruit reached an over-ripening and senescence stage in which quality attributes were considered as not optimum for consumption. Weight loss increased during storage in both cherry cultivars, either in fruit from control trees as from treated trees, although they were delayed in the last ones. Thus, for ‘Prime Giant’ weight losses in control fruit reached values of 10.86 ± 0.36% after 21 days of storage while significantly (*p* < 0.05) lower values, ≈7% were reached in fruit from melatonin control trees independently of the applied dose. For ‘Sweet Heart’ cultivar, weight losses were also significantly lower (*p* < 0.05) and dose dependent in fruit from melatonin treated trees than in controls, the lowest weight losses being found for 0.5 mM dose, with values of 6.40 ± 0.23% after 28 days as compared to 9.39 ± 0.16% in controls (Figure 1). Colour index (a*/b*) at harvest was significantly increased (*p* < 0.05) as a consequence of melatonin treatments with respect to controls in both cultivars and a similar trend was observed during storage, with increases during the first 1–2 weeks and decreases thereafter, except for ‘Prime Giant’ from 0.5 mM melatonin treated fruit, in which no changes occurred from day 0 to day 14 of storage (Figures 2A,C). However, colour index showed higher values, 9.21, 7.77, and 11.63%, in fruit from 0.1, 0.3, and 0.5 mM treated trees, respectively, for ‘Prime Giant’ and 2.46, 7.86, and 8.48% for ‘Sweet Heart’ than in controls taking into account data from all sampling dates. Fruit firmness was also found at significantly higher levels (*p* < 0.05) in fruit from melatonin treated trees than in controls at harvest and these differences were maintained during the whole storage period, in spite of the firmness decreases observed in all fruit for both cultivars (Figures 2B,D). At harvest, the highest effect on fruit firmness was observed for 0.3 and 0.1 mM melatonin doses in ‘Prime Giant’ and ‘Sweet Heart,’ respectively. However, during storage, no significant differences were observed between melatonin doses, with 25–30 and 15–20% higher firmness levels in treated fruit than in controls for ‘Prime Giant’ and ‘Sweet Heart’ cultivars, respectively, taking into account data of all sampling dates.

**FIGURE 3:**
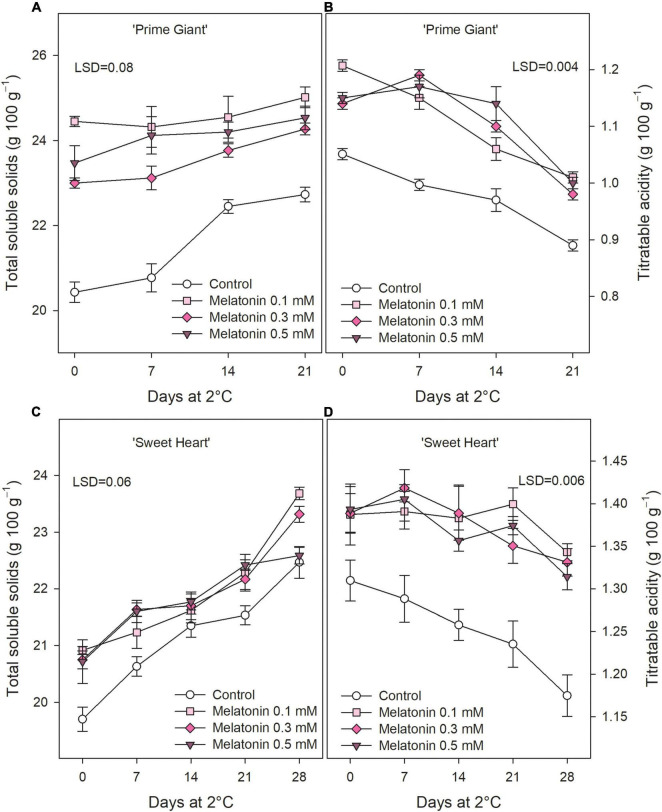
Total soluble solids **(A)** and titratable acidity **(B)** for ‘Prime Giant’ and ‘Sweet Heart’ **(C,D)** cultivars during storage at 2°C as affected by melatonin pre-harvest treatments. Data are the mean ± SE of three replicates. LSD values at (*p* < 0.05) are shown in each figure.

Total soluble solids in control fruit at harvest were 20.43 ± 0.24 and 19.70 ± 0.21 g 100 g^–1^ for ‘Prime Giant’ and ‘Sweet Heart,’ respectively, and significant increases (*p* < 0.05) occurred during storage (Figures 3A,C). Melatonin pre-harvest treatments led to significant (*p* < 0.05) enhanced TSS concentrations at harvest, the highest effects being observed for 0.1 mM in ‘Prime Giant’ (24.45 ± 0.12 g 100 g^–1^), while no significant differences among doses were observed for ‘Sweet Heart’ cultivar (≈21 g 100 g^–1^). Nevertheless, it is worth noting that TSS was higher in fruit from melatonin treated trees than in controls during the whole storage period. TA at harvest was 1.05 ± 0.01 and 1.31 ± 0.02 g 100 g^–1^ in control fruit of ‘Prime Giant’ and ‘Sweet Heart’ cultivars, respectively, and significant decreases (*p* < 0.05) occurred during storage. However, in fruit from melatonin treated trees, TA losses were delayed with respect to controls in both cultivars, and significantly (*p* < 0.05) higher values, ca. 15 and 10%, were observed as a consequence of melatonin treatments, either at harvest or during storage, in ‘Prime Giant’ and ‘Sweet Heart’, respectively (Figures 3B,D).

### Antioxidant Compounds and Antioxidant Enzymes

**FIGURE 4:**
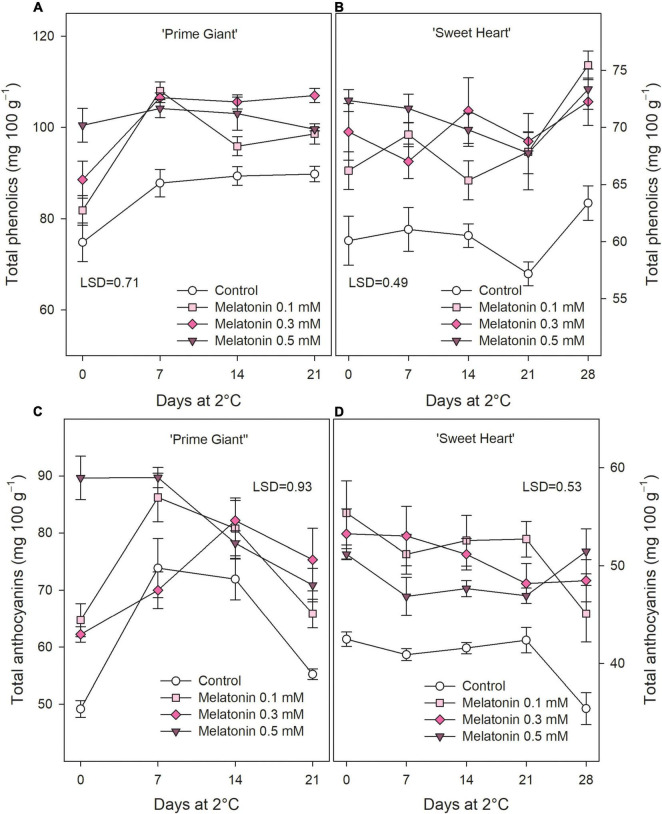
Total phenolics **(A,B)** and total anthocyanins **(C,D)** for ‘Prime Giant’ and ‘Sweet Heart’ cultivars during storage at 2°C as affected by melatonin pre-harvest treatments. Data are the mean ± SE of three replicates. LSD values at (*p* < 0.05) are shown in each figure.

Total phenolic concentrations at harvest was significantly increased by melatonin treatments in a dose-dependent way, from 74.84 ± 4.23 mg 100 g^–1^ in fruit from control trees to 100.48 ± 3.67 mg 100 g^–1^ in those from 0.5 mM treated ones, in ‘Prime Giant’ and from 60.08 ± 2.14 to 72.36 ± 0.96 mg 100 g^–1^ in ‘Sweet Heart.’ During storage, total phenolics were maintained at higher levels in fruit from melatonin treated trees than in controls, although no significant differences among melatonin doses were observed (Figures 4A,B). With respect to anthocyanin concentration, significant enhanced (*p* < 0.05) values were also found, in general, as a consequence of melatonin treatments, either at harvest or during storage, for both cultivars (Figures 4C,D). Taking into account data of all sampling date, total phenolic and anthocyanin concentration was ca. 25% lower in ‘Sweet Heart’ than in ‘Prime Giant,’ while the increase in concentrations of these bioactive compounds by melatonin treatments was higher in ‘Sweet Heart’ than in ‘Prime Giant.’

**FIGURE 5:**
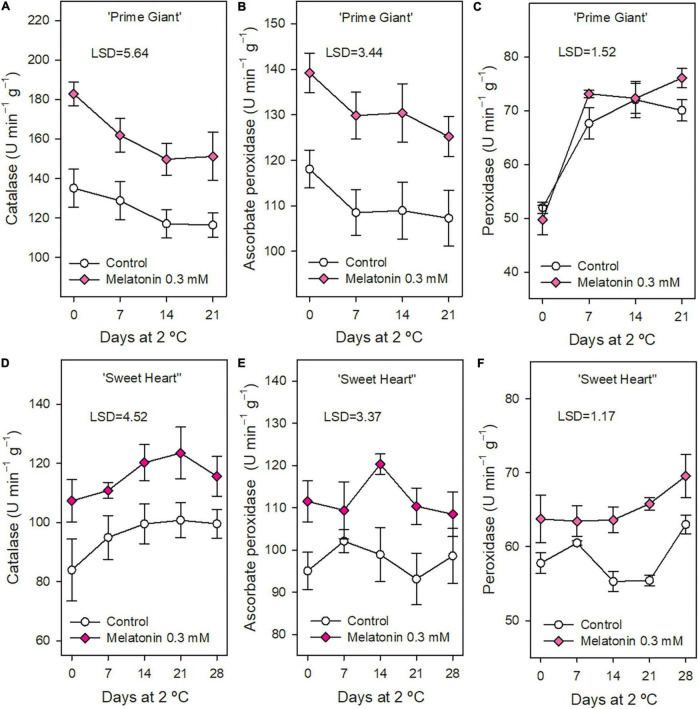
Catalase, ascorbate peroxidase, and peroxidase activities in ‘Prime Giant’ (**A–C**, respectively) and ‘Sweet Heart’ (**D–F**, respectively) cultivars during storage at 2°C as affected by melatonin pre-harvest treatments. Data are the mean ± SE of three replicates. LSD values at (*p* < 0.05) are shown in each figure.

In general, the activity of antioxidant enzymes CAT, APX, and POD was significantly higher (*p* < 0.05) in fruit from 0.3 mM melatonin treated trees than in controls, either at harvest or during storage, except POD activity in ‘Prime Giant’ cultivar (Figure 5). The highest effects were found for CAT activity, which was 30 and 20% higher in treated fruits for ‘Prime Giant’ and ‘Sweet Heart,’ respectively, during the whole storage period, while ≈15% increases were observed for APX activity in both cultivars.

## Discussion

Sweet cherry fruit quality traits, such as absence of visual defects, fruit size, colour, stem freshness, and length, firmness, aroma, flavour, sweetness, and sourness are the major responsible for consumer purchase decisions, although important differences have been reported among cultivars (Díaz-Mula et al., 2009; Serradilla et al., 2012; Correia et al., 2017). However, these quality parameters evolved quickly during fruit storage, even if storage is performed at appropriate temperature, leading to fruit with no optimal quality for consumption (Serrano et al., 2009; Chockchaisawasdee et al., 2016; Giménez et al., 2017; Zhang et al., 2021). These changes are mainly related to softening, losses of fruit weight and TA, and increases in TSS and colour as well as to fruit decay and browning and desiccation of the pedicel. Accordingly, the present results show increases in weight loss, TSS and colour and decreases in fruit firmness and TA, although these changes were significantly delayed in fruit from melatonin treated trees with respect to controls (Figures 1–3). Firmness and TA maintenance as a consequence of pre-harvest melatonin treatments are major factor contributing to preserve fruit during storage, since cherries with higher firmness are much appreciated by consumers and TA retention during storage led to cherries with the aroma and taste of recently harvested cherries (Valero et al., 2011; Díaz-Mula et al., 2012; Serradilla et al., 2012).

Thus, taking into account all these quality parameters, storage time with optimal fruit quality properties for consumption in control cherries for both cultivars was 14 days, while it could be extended up to 21 and 28 days in fruit from melatonin treated trees for ‘Pime Giant’ and ‘Sweet Heart’ cultivars, respectively. Accordingly, post-harvest ripening was delayed in ‘Guifei’ mangoes by 0.5 mM melatonin dipping treatment for 1 h (Liu et al., 2020) and in banana in a concentration dependent manner in the range of 0.05–0.5 mM (Hu et al., 2017), leading to extension of fruit shelf life. Similar results have been reported in peaches (Gao et al., 2016), and nectarines (Bal, 2021), and these effects were attributed to inhibition of ethylene production in those climacteric fruit species. Maintenance of fruit quality traits and extension of shelf-life seem to be general fruit responses to melatonin post-harvest dipping treatments since they have been also reported in non-climacteric fruit, such as pomegranate and strawberry as recently revised by Ze et al. (2021). Specifically, in ‘Sunburst’ sweet cherries, post-harvest 0.05, 0.1, and 0.15 Mm melatonin treatments led to delay the post-harvest ripening process (Wang et al., 2019) and in ‘Siah Mashhad’ cultivar dipping with 0.001, 0.01, 0.1, and 1 mM melatonin reduced flesh browing and decay after 45 days of storage, the highest effect being found with 0.1 mM dose (Sharafi et al., 2021). However, post-harvest fruit treatments have consumers’ concerns and legal restrictions and then, there is a need of research regarding pre-harvest treatments with effect on fruit quality properties at harvest and during storage. In this sense, pre-harvest treatments of apricot tree with melatonin increased fruit quality parameters at harvest and these quality traits were maintained during storage (Medina-Santamarina et al., 2021). Higher values of quality parameters, either at harvest or during storage, were observed on pomegranate fruit as a consequence of melatonin tree treatments during on-tree fruit development (Lorente-Mento et al., 2021). In sweet cherry, 0.05, 0.1, and 0.2 mM melatonin applied on tree canopy (3, 2, and 1 weeks before harvest) resulted in fruit with higher TSS and lower TA in the ‘Hongdeng’ cultivar (Xia et al., 2020), but no storage experiment was performed in this research. On the contrary, similar treatments with 0.5 mM melatonin did not show significant effect on ‘Ferrovia’ fruit quality parameters at harvest and softening was the only parameters related to fruit quality and senescence delayed after 14 days of cold storage (Michailidis et al., 2021). Thus, the effects of pre-harvest melatonin treatment on delaying fruit ripening and senescence will be different depending on cultivar, applied concentration or fruit developmental stage, among other factors.

In the last decade, special attention has been paid to the content on bioactive compounds with antioxidant activity, such as anthocyanins and other phenolic compounds, in sweet cherry due to their positive impact on human health, by reducing the risk of suffering from several degenerative diseases (Correia et al., 2017; Gonçalves et al., 2018, 2019; Antognoni et al., 2020; Faienza et al., 2020; Luo et al., 2021). In this fruit species, the red colour intensity is due to their content of anthocyanins and their profile, the major anthocyanin being cyanidin 3-*O*-rutinoside comprising around 90% of total anthocyanins, and 70% of total phenolic compounds, in most of the studied cultivars, including ‘Prime Giant’ and ‘Sweet Heart’ (Usenik et al., 2008; Serrano et al., 2009; Martínez-Esplá et al., 2014; Antognoni et al., 2020; Gonçalves et al., 2021; Carrión-Antolí et al., 2022). Total phenolic concentration showed an upward trend from day 0 until the end of storage in fruit from control and treated trees for both cultivars (Figures 4A,B), while total anthocyanins, generally, increased during the first weeks of storage and decreased thereafter (Figures 4C,D). These results are in agreement with previous reports in other cherry cultivars, which have been related to the ongoing ripening process after harvesting (Serrano et al., 2009; Valero et al., 2011; Giménez et al., 2014; Sharafi et al., 2021). However, it is wort noting that phenolic and anthocyanin contents at harvest were enhanced as a result of melatonin treatments and maintained at higher levels in treated fruit than in controls during storage (Figures 4A–D). Accordingly, post-harvest melatonin dipping treatments have been reported to increase phenolic and anthocyanin concentrations during storage in some fruit species, such as strawberry (Liu et al., 2018), tomato (Sharafi et al., 2019), and pomegranate (Aghdam et al., 2020) and even in sweet cherry, as has been recently reported by Sharafi et al. (2021). These effects were attributed to melatonin stimulation of the phenylpropanoid pathway mainly by enhancing phenylalanine ammonialyase and chalcone synthase activities. However, literature regarding the impact of pre-harvest melatonin treatments on phenolic and anthocyanin evolution during storage is scarce.

In sweet cherry, the effects of pre-harvest melatonin treatments on anthocyanins and phenolic content have been reported only in three previous papers and contradictory results are observed. Thus, higher total phenolic and anthocyanin contents at harvest in ‘Hongdeng’ cultivar were found after tree treatment with 0.05 and 0.1 mM melatonin 3, 2, and 1 week before harvest (Xia et al., 2020). On the contrary, fruit treatment of ‘Prime Giant’ cultivar at stage II with 0.1 mM did not show impact on anthocyanin content at harvest while 0.01 mM dose led to twofold lower anthocyanin concentration as compared with control (Tijero et al., 2019). In ‘Ferrovia’ cultivar, 0.5 mM melatonin treatments, 2 and 1 week before harvest, had no effects on individual phenolic or anthocyanin compounds at harvest (Michailidis et al., 2021). However, as far as we know, only Michailidis et al. (2021) have reported the effects of cherry tree pre-harvest melatonin treatment on these bioactive compound evolution during storage and showed higher levels of neochlorogenic acid and cyaniding 3-*O*-rutinoside (the major phenolic and anthocyanin, respectively) after 12 days of cold storage in fruit from treated trees than in controls.

Fruit ripening and senescence are associated with ROS accumulation, such as H_2_O_2_, O_2_^–•^ and OH^–•^, which are involved in DNA and proteins damage, peroxidation of membrane lipids and acceleration of senescence processes (Hodges et al., 2004). These ROS are generated in normal metabolism of plant cells and scavenged by antioxidant compounds (such as phenolics, tocopherols, carotenoids, and ascorbic acid) and by antioxidant enzymes, mainly superoxide dismutase (SOD), POD, CAT, and APX contributing to repair cell oxidative damage (Hodges et al., 2004; Kumar et al., 2014). Antioxidant enzymes were measured in fruit from control and 0.3 mM treated trees, since, in general, similar effects on maintaining cherry quality properties were observed for 0.3 and 0.5 mM concentrations, as well as on increasing crop yield (Carrión-Antolí et al., 2022). The results of the present study show higher activities of these antioxidant enzymes in melatonin treated trees than in controls during the whole storage period in both cultivars (Figures 5A–F). Thus, the occurrence of increased activity of antioxidant enzymes and enhanced content of the antioxidant compounds, phenolics and anthocyanins, could be responsible for the delay in the fruit post-harvest ripening process and maintenance of fruit quality attributes observed in sweet cherries from melatonin treated trees. Accordingly, different post-harvest treatments aimed to delay the sweet cherry post-harvest ripening and senescence processes also increased these antioxidant enzymes during storage. Thus, chitosan coating enhanced CAT and POD activities (Dang et al., 2010), as well as vacuum cooling treatment before storage (He et al., 2013) and 1-methylcyclopropene and hexanal increased SOD activity and reduced decreases in APX activity during storage compared to control cherries (Sharma et al., 2010). Nano-silica-chitosan solution and pressurised Argon treatment, and specially the combination of both treatments, led also to increased activities of CAT, APX, SOD, POD, and glutathione reductase (GR) and reduced accumulation of H_2_O_2_ and O_2_^•–^ during sweet cherry storage as compared with controls, resulting in fruit with delayed senescence and extended shelf life (Meng et al., 2022). Pre-harvest sweet cherry treatments with SA, ASA, and SaMe led also to higher activities of CAT, POD, APX, and SOD and increased concentrations of phenolics and anthocyanins in treated fruit at harvest and during storage as compared with controls (Valverde et al., 2015; Giménez et al., 2017). Thus, treatments leading to increase sweet cherry ROS elimination systems, as observed in the present experiments for cherries from melatonin-treated trees, could contribute to delaying the post-harvest ripening and senescence processes and extending their shelf life. Accordingly, post-harvest melatonin treatment significantly induced enzymatic antioxidants and non-enzymatic antioxidants during storage in mango, kiwifruit, pomegranate, and peach fruit as reviewed by Xu et al. (2019) and Ze et al. (2021). The expression of genes encoding for antioxidant enzymes was upregulated by melatonin treatment, although the molecular mechanism underlying these effects needs further research. In sweet cherry, increased activity of antioxidant enzymes during storage due to post-harvest melatonin treatment has also been recently reported (Sharafi et al., 2021), although these effects due to melatonin applied as foliar spray treatment to sweet cherry trees have been reported for the first time in the present experiments.

## Conclusion

Overall results showed that melatonin treatments during sweet cherry fruit on-tree development reduced weight and TA losses, softening and changes in fruit colour and TSS during cold storage. In addition, total phenolic and anthocyanin concentrations were higher in fruit from treated trees than in those from control ones, either at harvest or during the whole storage period. Finally, the activity of the antioxidant enzymes CAT, APX, and POD was also enhanced as a consequence of melatonin treatment. Thus, the storage period of fruit with quality properties in optimum levels for consumption was extended by one and 2 weeks for ‘Prime Giant’ and ‘Sweet Heart’ cultivars, respectively, with respect to fruit from control trees. The increase of antioxidant systems, both enzymatic and non-enzymatic ones, as a consequence of melatonin treatments would lead to a more efficient ROS elimination accounting for delaying the post-harvest ripening process and maintaining fruit quality during storage.

## Data Availability Statement

The original contributions presented in the study are included in the article/supplementary material, further inquiries can be directed to the corresponding author.

## Author Contributions

DV and MS conceived and designed the work in association with other authors. AC-A performed field treatments and most of the analytical determination, in collaboration with DM-R, PJZ, FG, MS, and DV. MS and DV analysed the data and wrote the manuscript. DV and MS were responsible for funding acquisition. All authors contributed to review the article and approved the submitted version.

## Conflict of Interest

The authors declare that the research was conducted in the absence of any commercial or financial relationships that could be construed as a potential conflict of interest.

## Publisher’s Note

All claims expressed in this article are solely those of the authors and do not necessarily represent those of their affiliated organizations, or those of the publisher, the editors and the reviewers. Any product that may be evaluated in this article, or claim that may be made by its manufacturer, is not guaranteed or endorsed by the publisher.
